# HIV Assembly and Budding: Ca^2+^ Signaling and Non-ESCRT Proteins Set the Stage

**DOI:** 10.1155/2012/851670

**Published:** 2012-06-12

**Authors:** Lorna S. Ehrlich, Carol A. Carter

**Affiliations:** Department of Molecular Genetics & Microbiology, Stony Brook University, Life Sciences Building Room 248, Stony Brook, NY 11794-5222, USA

## Abstract

More than a decade has elapsed since the link between the endosomal sorting complex required for transport (ESCRT) machinery and HIV-1 protein trafficking and budding was first identified. L domains in HIV-1 Gag mediate recruitment of ESCRT which function in bud abscission releasing the viral particle from the host cell. Beyond virus budding, the ESCRT machinery is also involved in the endocytic pathway, cytokinesis, and autophagy. In the past few years, the number of non-ESCRT host proteins shown to be required in the assembly process has also grown. In this paper, we highlight the role of recently identified cellular factors that link ESCRT machinery to calcium signaling machinery and we suggest that this liaison contributes to setting the stage for productive ESCRT recruitment and mediation of abscission. Parallel paradigms for non-ESCRT roles in virus budding and cytokinesis will be discussed.

## 1. Focus of This Paper

Determinants intrinsic to the structural precursor polyprotein (Gag) that is encoded by the Human Immunodeficiency Virus-type 1 (HIV-1) and other retroviruses direct targeting of Gag to the plasma membrane, membrane and genome RNA binding, Gag multimerization, and budding of the assemblage into the extracellular space as virus particles (reviewed in [1–4]). Through a proteomic search aimed at identification of cellular factors that might participate with Gag and ESCRT, we identified the inositol 1,4,5-triphosphate receptor (IP3R) as a protein enriched in an endosome- and plasma-membrane-enriched fraction [5] only when Gag was expressed (unpublished observation). IP3R protein forms a transmembrane calcium ion (Ca^2+^) channel that is mostly found on the membrane of the endoplasmic reticulum (ER), the major intracellular Ca^2+^ store in the cell. IP3R has also been detected on the plasma membrane, late endosome/multivesicular bodies (LE/MVBs), and the nucleus (reviewed in [6–8]). Efficient HIV-1 Gag trafficking and viral particle release were shown to require activation of IP3R [9]. IP3R activation requires phospholipase-C- (PLC-) catalyzed hydrolysis of PI(4,5)P_2_ to generate inositol 1,4,5-triphosphate (IP3), the activating ligand for the receptor (reviewed in [6–8]). Binding of IP3 initiates conformational changes leading to channel opening and release of Ca^2+^ into the cytosol [10]. Earlier studies on HIV particle production had demonstrated that induction of a transient rise in the cytosolic Ca^2+^ concentration resulted in a dramatic rise in viral particle release, suggesting that Ca^2+^ is a limiting factor in late-stage replication [11, 12]. Taken together, these observations collectively suggested that IP3R is the physiological provider of the required Ca^2+^. The proteomic search also identified several additional proteins that function in regulation of Ca^2+^ signaling, including Sprouty2 (Spry2), a modulator of Ca^2+^ signaling [13] and other modes of signaling [14, 15]. We demonstrated that Spry2 is also required for productive HIV egress [16, 17]. Proteins such as IP3R and Spry2 have been shown to function with the same elements of cytoskeletal and vesicular transport that are integral to ESCRT machinery [18–20]. Over the past few years, a number of other non-ESCRT host proteins have been shown to be required for Gag assembly. Some of these have been discussed in recent reviews [2, 21, 22]. We will discuss how these host proteins set the stage for ESCRT recruitment and ESCRT-mediated abscission events. We apologize to those investigators whose studies may be pertinent but were not explicitly cited. 

## 2. Introduction 

Enveloped viruses, like HIV-1, exit the host cell by budding. The segment of the plasma membrane that serves as assembly platform evaginates to form the budded particle and becomes the viral envelope. Since the Gag precursor is the viral gene product that plays the key role in recruiting other viral components to the assembly site [23, 24], the assembly process must necessarily include a mechanism for stable localization of Gag at the plasma membrane (PM). Once on the PM, Gag has intrinsic assembly capability that is attributed to functions of its four domains (matrix-capsid-nucleocapsid-p6). The N-terminal matrix (MA) domain mediates membrane binding ([25–29] and references in [1]). The capsid (CA) domain provides Gag with capability for self-assembly into higher-order multimers ([30–35] and references in [36]). The nucleocapsid domain (NC) mediates binding to viral RNA and nonspecific RNAs as well as promoting Gag association [37–39] and references in [40]. The C-terminally located p6 region mediates the untethering of the assembled Gag particle from the host [41, 42]. Orderly cleavage of Gag at interdomain junctions within the structural precursor polyprotein by a virus-encoded proteinase [43–47] occurring concurrently with budding results in mature proteins whose rearrangement transforms the bud to a mature, infectious particle [48, 49]. The final step of the virus assembly process, which results in the pinching off of the particle from the host cell, is mediated by ESCRT proteins that have been recruited to the bud neck by motifs in p6 that are designated as “late” or L domains (reviewed in and references in [50, 51]). Thus, Gag is both necessary and sufficient for viral particle assembly [52]. 

## 3. Plasma Membrane Targeting: Role of PI(4,5)P_**2**_

As a cytosolic protein, the synthesis of Gag takes place on soluble polysomes in the cell interior [53]. A myristoylation reaction occurs cotranslationally during which Gag acquires a myristoyl moiety on the N-terminal glycine which plays a role in assembly [28, 54, 55]. At the earliest experimentally feasible time points, Gag has been demonstrated to have a cytosolic distribution when examined by confocal microscopy [11], biochemical fractionation [56], and immunogold electron microscopy [57]. Eventually, the entire Gag population becomes membrane associated with the PM as the preferred site at steady state (references in [23]). This is consistent with the results of *in vitro* binding studies wherein MA, which is highly basic ([25–29] and references in [1]), mediates binding to membranes reconstituted with acidic phospholipids ([26, 27] and references in [1]). It is also consistent with observations that the cytoplasmic leaflet of the PM is unique among cell membranes in having a net negative charge due to high levels of acidic phospholipids [58]. The targeting phospholipid was identified as the complex acidic phospholipid, phosphatidylinositol 4,5 bis-phosphate (PI(4,5)P_2_) [59]. Depletion of PI(4,5)P_2_, using plasmamembrane-targeted lipid phosphatases, caused Gag to be localized to LE/MVBs and prevented Gag localization to the PM [59]. PI(4,5)P_2 _ is mostly found on the PM where it represents a minor plasma membrane lipid component [60]. Structural analysis of PI(4,5)P_2_ binding to HIV-1 MA shows contacts made by the head group (i.e., phosphates and inositol ring) with basic residues and the nestling of adjacent acyl groups into a hydrophobic cleft [61] while studies with full-length Gag underscored the importance of the phosphoinositide acyl chain [62]. These *in vitro* studies also predict initiation of Gag structural changes following PI(4,5)P_2_ binding. Studies with the matrix protein show that PI(4,5)P_2_ binding results in exposure of the N-terminal myristate [61]. Studies with Gag in the presence of nucleic acid reveal an interplay between binding to PI(4,5)P_2_, binding to nucleic acid, and capsid (CA) domain-mediated self-association [63]. The model of Gag membrane association founded on Gag interaction with PI(4,5)P_2_ is supported by the inhibitory effect on Gag particle release of depletion of plasma membrane PI(4,5)P_2_ [59, 64, 65]. It should be noted that as important as PI(4,5)P_2_ is to HIV-1 Gag membrane targeting, the importance of PI(4,5)P_2_ to targeting and release of other retroviral Gags varies. Mo-MLV exhibits a preference and a requirement for PI(4,5)P_2_ [66]. Equine infectious anemia virus (EIAV) budding is less impacted by depletion of PI(4,5)P_2_ due to preferential binding to PI(3,5)P_2_ [65]. PI(3,5)P_2_ is a phospholipid that is predominantly associated with endosomal compartments at steady state [67] implying endosomal targeting of EAIV Gag in the cell. EIAV Gag trafficking requires such targeting as inactivation of the PI(3)P_2_ 5-kinase, which is responsible for the endosomal placement of PI(3,5)P_2_ [67], inhibits EIAV Gag VLP production [65]. ASV budding appears to rely on electrostatic interaction with acidic phospholipids and exhibits no specific reliance on phosphoinositide components of the PM [68]. Thus, HIV-1 Gag membrane association is mediated by a specific bipartite determinant in the MA domain comprised of myristate and basic amino acid clusters [1] with Gag-PI(4,5)P_2_ binding serving as the basis for targeted membrane association. Gag's preferential association with the plasma membrane is due to two inherent features of PI(4,5)P_2_: (i) the PM is where most of cellular PI(4,5)P_2_ is located [60] and (ii) PI(4,5)P_2_ molecules are products of *in situ* synthesis (i.e., PM-localized molecules are produced at the PM; [69]). Thus, PI(4,5)P_2_ targeting provides a mechanism to direct Gag from its site of synthesis in the cell interior to the plasma membrane. 

Detection of assembled HIV-1 Gag inside membrane compartments with the characteristics of LE/MVBs has been documented [70, 71], and altered Gag residency in LE/MVBs following stimulatory or inhibitory effects on virus production has been demonstrated [11, 72, 73]. Additionally, the virus particle has components that are typical exosome markers [74]. However, for macrophages, at least, those apparently intracellular membrane compartments with LE/MVB features were demonstrated to be actually extracellular space delineated by intracytoplasmic plasma membrane [75, 76]. Moreover, Gag particle production has been shown to be insensitive to interference with LE/MVB function [77]. The role of the LE/MVB in Gag assembly and release thus remains controversial. We suspect that at the root of this controversy is the complex nature of the LE/MVB itself. It cannot be precluded that the endosomal machinery can interact with Gag in the traditional manner, wherein ESCRT machinery facilitates sorting of cargo proteins into MVBs for ultimate delivery to degradative compartments. However, the handling of sorted proteins by the MVB is not always unidirectional. Though targeted to the LE/MVB in both HeLa and Jurkat cells, the 29KE/31KE Gag mutant is released at near wild-type levels from Jurkat cells but is trapped inside HeLa cells [78] which shows that trafficking within the MVB can be influenced by its environment (i.e., cell dependence). EIAV Gag is another interesting case since, despite its endosomal targeting, EIAV Gag VLPs are released from cells such as COS-1 and HeLa [65]. It would be interesting to know if EIAV Gag induces any alteration in the MVB and, if so, whether this facilitates productive infection. Direct delivery of Gag to the site of release on the plasma membrane circumvents the potentially nonproductive outcome of Gag association with endosomal machinery. A Gag assembly model that incorporates Gag-PI(4,5)P_2_-based targeting of Gag to assembly sites on the PM permits a more productive path from Gag synthesis to release of an assembled Gag particle. 

## 4. Late Domains in Gag Recruit ESCRT Machinery 

Budding structures accumulate on the plasma membrane if the C-terminal p6 region is missing from Gag [41, 42]. The p6 region bearing the L domain has counterparts in other retroviruses and is functionally exchangeable with these within and outside the genera; for example, the PTAP motif from the p6 region of HIV-1 Gag was shown to substitute for the PY motif in the L domain-bearing region (p2b) of the avian sarcoma virus (ASV) and vice versa [79–83] and references in [50, 51, 84, 85]. Functional exchangeability demonstrates that there are multiple, though not necessarily equally effective, ways for Gag to access the ESCRT machinery. Accordingly, Tsg101 as binding partner of the HIV PTAP motif and Nedd4 family members as binding partner of the ASV PY motif facilitate release of HIV-1 and ASV, respectively, through functionally exchangeable but independent routes (i.e., Tsg101 can replace Nedd4 function in facilitating ASV budding [86, 87]). Members of the Nedd4 family of ubiquitin ligases can also replace Tsg101 in facilitating HIV-1 release under certain circumstances [88–91]. The binding of the ESCRT adaptor, Alix, to the secondary L domain in Gag serves this purpose as well (reviewed in [92]). The ESCRT machinery is now known to comprise >25 proteins, organized into four complexes (ESCRT-0, -I, -II, and -III) that function sequentially along with several additional associated factors (reviewed in [93–95]). Irrespective of how Gag is linked to the ESCRT machinery, in all cases ESCRT-III and Vps4 must be recruited to the bud neck at the membrane site to execute the final bud scission event and to release the ESCRT factors from the assemblage for recycling back to the cytosolic pool for participation in future events [96, 97]. A feature of retroviral utilization of the ESCRT machinery is the selective use of the ESCRT complexes. HIV-1 viral particle production requires ESCRT-I and ESCRT-III but not ESCRT-II [98] while ASV requires ESCRT-II but not ESCRT-1 [99]. These observations, along with recognition that ESCRTs, which normally function in transport of some cellular proteins to degradative cellular compartments, are required for exit of assembled Gag from the cell, suggests that non-ESCRT host proteins may play a key role in allowing the ESCRT machinery to be utilized differentially by the virus compared to the host. Thus, non-ESCRT proteins may permit HIV to exploit ESCRT machinery by preventing the Gag-ESCRT complex from participating in interactions with ESCRT partners that are nonproductive for the virus. 

## 5. Parallels between HIV-1 Budding, Cytokinesis, and Autophagy

*“All organisms do things the same way except that it is completely different in every detail” J. Haber*

The abscission event in virus budding results in separation of the enveloped virus from the host cell. Another process where the abscission event results in separation of two membrane-enclosed cellular entities is cytokinesis. Cytokinesis, itself a multistep process, is the terminal stage in cell division [100]. Abscission of the intercellular bridge/midbody results in separation of the mitotic daughter cells. Recruitment of ESCRT and mediation of the abscission event by ESCRT is the basis for the parallel between HIV-1 budding and cytokinesis [101, 102]. The parallel may extend to events occurring before ESCRT recruitment and participation, (i.e., in a pre-ESCRT stage). Paradigms that govern the pre-ESCRT stage of cytokinesis, which has been an active area of research long before discovery of HIV, may likewise apply to the pre-ESCRT stage of viral budding. 

**Figure 1:**
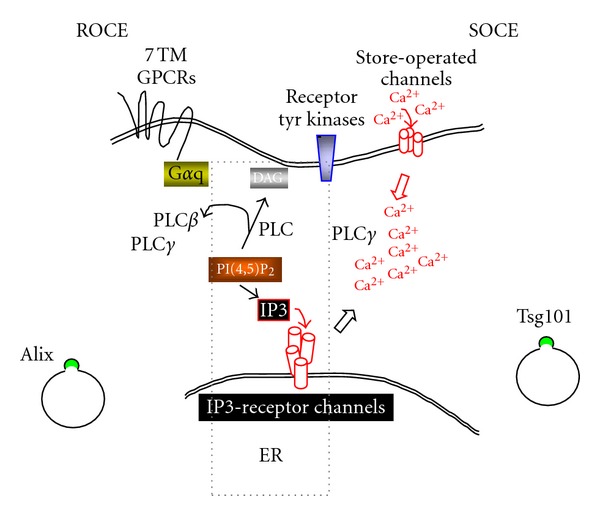
Elements of Ca^2+^ signaling machinery implicated in HIV-1 release. Tsg101-mediated release requires the core elements, IP3R, PI(4,5)P_2_, and PLC. Alix-mediated release requires these, SOCE and ROCE. It is not known whether SOCE and ROCE are controlled by distinct Ca^2+^ channels [120] or if the same channel complexes mediate SOCE when recruited to lipid rafts and ROCE when they are outside of lipid rafts [121].

A theme that is emerging as a cell prepares for cytokinesis is the reshaping of calcium signaling [103]. Local and global elevations in cytosolic Ca^2+^ level are achieved by ion release from the ER (the cell's major intracellular Ca^2+^ store) and by influx from the extracellular environment [104]. Decrease in Ca^2+^ content of the ER triggers activation of Ca^2+^ influx channels on the plasma membrane and refilling of the ER store in a process called store-operated-calcium-entry (SOCE) [105, 106]. A major cellular change that occurs during cell division prior to cytokinesis is the uncoupling of Ca^2+^ store depletion and SOCE [107, 108]. Why this is necessary is presently not known but the effect is to render the pre-ESCRT events in cytokinesis independent of SOCE and reliant on the internal stores as the Ca^2+^ source. Independence from SOCE and reshaping of calcium signaling as a pre-ESCRT stage paradigm also appear to be the case for HIV-1 budding. Blockade of SOCE with 2-aminoethoxydiphenylborate (2-APB), a small molecule inhibitor of store refilling through SOCE [109], had no effect on release of the HIV-1 Gag particle [110]. Blockade of a G protein-coupled receptor cascade [111] triggered by Ca^2+^ entry through receptor-operated calcium entry (ROCE; [112]) also had no effect on Gag particle release [110]. Additionally, cells where productive Gag budding is occurring (i.e., expression of wild-type Gag) exhibit higher cytosolic Ca^2+^ compared to mock-transfected cells or cells expressing a budding-impaired PTAP Gag mutant [110]. Possibly, insulating the calcium machinery from external Ca^2+^ sources allows both virus budding and cytokinesis to proceed more efficiently.  Figure 1 shows the elements of the Ca^2+^ signaling machinery implicated in HIV-1 release.

**Figure 2:**
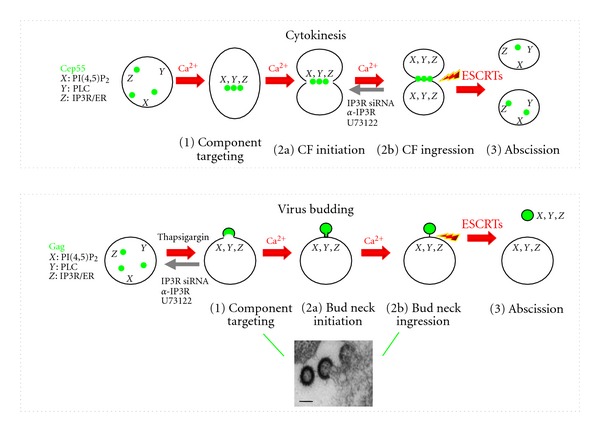
Similarities between cytokinesis (top) and viral particle production (bottom). CF: cleavage furrow. EM image shows HIV-1 VLPs in the process of budding. Bars indicate 100 nm.

Cytokinesis and viral budding share several general features (Figure 2). The first step in both processes is the targeting of the requisite components to the eventual scission site, that is, the plasma membrane. Formation of the cleavage furrow is a visual marker of initiation of cytokinesis and aspects of this event that appear similar to the budding process are furrow ingression, that is, a progressive narrowing of the eventual scission region to form a bud neck. In cytokinesis, the separating bodies are of comparable volumes; in viral budding, they are of unequal volumes. IP3R, intact PI(4,5)P_2_, PI(4,5)P_2_ hydrolysis, and Ca^2+^ are all required for the normal progression of cytokinesis in cellular systems where cell division has been well studied, for example, spermatocyte and oocytes [113–116]. There is a requirement for Ca^2+^ to maintain furrow or neck stability, necessitating constant PLC-mediated hydrolysis of PI(4,5)P_2_ [117, 118]. Components involved in Ca^2+^ mobilization and cytoskeleton remodeling are recruited to the furrow [117–119]. Similarly, in addition to intact PI(4,5)P_2_ [59], HIV budding requires IP3R and PLC activity [9, 110]. Analogous to IP3R recruitment to the furrow in cytokinesis, there is also recruitment of IP3R to Gag budding sites on the plasma membrane [110]. 

In cytokinesis, the non-ESCRT protein mediating recruitment of ESCRTs is Cep55. Cep55 recruits Tsg101, a component of ESCRT-I, and Alix, an ESCRT adaptor protein that binds both ESCRT-1 and ESCRT-III, to the eventual scission site once furrow ingression is completed [101, 102, 122–124]. These ESCRT factors, in turn, recruit the ESCRT-III complex required to carry out the terminal step in cytokinesis, abscission, that is, the severing of the thin intercellular bridge that connects the two daughter cells [125–127]. The counterpart of the Cep55-ESCRT link in viral budding is the targeting of Gag to the eventual scission site on the plasma membrane and recruitment of Tsg101 and/or Alix through the L domains and eventually ESCRT-III.

Autophagy, the process involved in the breakdown of intracellular proteins and organelles, is now appreciated as a mechanism of great importance in both cell survival and cell death [128]. It is the latest cellular process linked to ESCRT function. Indeed, autophagy is a necessary postabscission step in cytokinesis [129]. Following cytokinesis, the dividing cells are connected by an intracellular bridge that contains the midbody. This structure persists long after division as a midbody derivative that is inherited asymmetrically by the daughter cell with the older centrosome. Recent findings in mammalian cells and in *Drosophila melanogaster* indicate that ESCRTs are required for efficient trafficking through the endolysosomal system where the autophagic cargo is degraded [130–132]. As with cytokinesis and viral budding, IP3R-mediated Ca^2+^ signaling is emerging as critical for the pre-ESCRT stage in autophagy [133]. *De novo* synthesis of phospholipids is coupled with autophagosome formation [134]. Pairing phosphoinositides with Ca^2+^ ions in endolysosomes has been suggested to control the direction and specificity of membrane trafficking [135]. All three processes, cytokinesis [136], viral budding [137], and autophagy [138, 139], require or involve SNAREs to conduct some of the critical events. The participation of calcium machinery components in all three processes suggests that the requirement for and reshaping of calcium signaling is a common feature governing their pre-ESCRT stages. 

## 6. Non-ESCRT Proteins and Other Factors Engaged in the Pre-ESCRT Stages of HIV-1 Assembly

For a number of non-ESCRT host proteins shown to be important for release of the Gag particle [2, 4, 22, 140], disruption of the protein function does not result in the canonical L domain phenotype (i.e., arrested budding structures at the periphery of cells examined by EM). Rather, Gag is found in the cell interior. We and others [2] interpret this to indicate participation of these proteins in assembly step(s) preceding ESCRT-mediated budding. Some of these proteins have regulatory links to each other. Among these are the human vacuolar protein sorting (hVps) protein 18 (Vps18), a class C Vps complex component, and Mon2. Both have been shown to be required for Gag PM localization and virus production [141]. In yeast, class C Vps proteins have been shown to regulate PM localization of at least one protein [142] and to assume roles antagonistic to ESCRT in the recycling of membrane proteins [143]. The human orthologue of Mon2 (hMon2) can bind and regulate the subcellular localization of adaptor proteins such as AP-1, AP-3, and Arf1 which have previously been shown to be required for Gag PM localization and Gag particle production [72, 144, 145]. The notion of non-ESCRT proteins regulating the activity of other non-ESCRT proteins in the pre-ESCRT stage has a parallel in cytokinesis as illustrated by the host protein, TEX14. This non-ESCRT protein binds Cep55 at the same motif used to recruit Tsg101 or Alix and negatively regulates ESCRT recruitment [146]. Through protein-protein interactions, non-ESCRT proteins could thus impose temporal and spatial control of the recruitment of participating proteins, including Gag itself, to assembly sites on the PM during the pre-ESCRT stage. 

**Figure 3:**
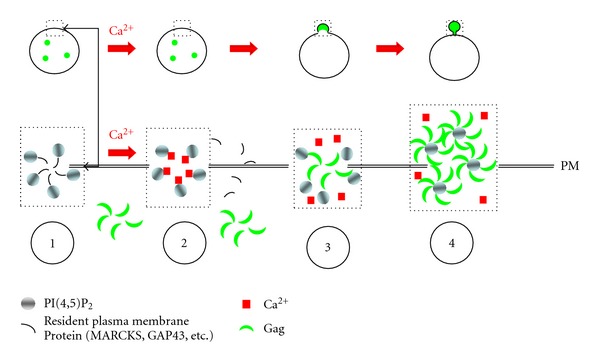
Ca^2+^ facilitates Gag-PI(4,5)P_2_ interaction and stabilization on the plasma membrane. Top, the squares highlight the top-down view of the plasma membrane shown below. Bottom, (1) most of the PI(4,5)P_2_ on the plasma-membrane is sequestered with plasma membrane-resident proteins that are highly basic and therefore unavailable to Gag. (2) A local rise in Ca^2+^ permits the cation to replace the resident proteins, freeing the PI(4,5)P_2_ from these proteins. (3) PI(4,5)P_2_, made available by Ca^2+^, recruits Gag to the plasma membrane. (4) Gag multimerization forms local PIP2 clusters that stabilize Gag association with the membrane, preventing loss of Gag from the narrowing bud neck in preparation for ESCRT recruitment.

Another pre-ESCRT event is alteration of the lipid composition of the assembly site. Quantitative analyses indicate that the viral envelope differs from the PM of its host cell in having higher levels of cholesterol and PI(4,5)P_2_ [58, 147]. Since the viral envelope is derived from the PM microdomain serving as the Gag assembly site, reorganization of the lipid bilayer in this location may occur as part of the assembly process. A feature of PM PI(4,5)P_2_ is that the greater majority is sequestered by electrostatic interaction with basic proteins that are resident at the PM (e.g., myristylated alanine-rich C kinase substrate (MARCKS; growth-associated protein (GAP)43; N-methyl-D-aspartate (NMDA) receptor, and the epidermal growth factor receptor (EGFR)) and is only released by a local rise in Ca^2+^ [148]. Another property of PI(4,5)P_2_ is that it does not have a natural inclination for clustering due to the energy barrier posed by repulsion of the large polar head groups when they are in proximity. It has been shown that Ca^2+^ can reduce this barrier and induce PI(4,5)P_2_ clustering in lipid monolayers [149]. Recruitment of IP3R machinery to the cell periphery and release of Ca^2+^ may function to increase the portion of PM PI(4,5)P_2_ available for interaction with Gag and to permit the clustering of PI(4,5)P_2_ molecules upon Gag multimerization. This model is summarized in Figure 3 and may explain how the budding requirement for both intact and hydrolyzed PI(4,5)P_2_ could be simultaneously resolved.

That budding structures are still formed by Gag mutants with disrupted PTAP motifs despite their impairment in recruitment of Tsg101 or in cells where Tsg101 has been depleted [50, 51] indicates that assembly site membrane deformation is a pre-ESCRT stage event. Although not required for initiation [116], Ca^2+^ is required for furrow ingression and for stability of the intercellular bridge in cytokinesis [113–116]. Furrow ingression in the presence of Ca^2+^ leads to a productive ESCRT recruitment stage as indicated by completion of cytokinesis. Analogous to furrow ingression is the formation of the virus bud neck where the ESCRT scission complex is recruited. The fact that the budding structures of Gag mutants with disrupted PTAP motifs accumulate on the plasma membrane indicates a failure in ESCRT recruitment even though the mutant has been demonstrated to be capable of employing alternative modes of linking to ESCRT (i.e., via Nedd4 or Alix). Our study [110] shows that, in cells expressing HIV-1 Gag, IP3R was translocated from the cell interior to the periphery and colocalized with Gag on the plasma membrane. Interestingly, IP3R redistribution is not induced in cells expressing the PTAP Gag mutant even though release of the mutant, albeit inefficient, also requires IP3R-regulated machinery. The lack of Ca^2+^ store recruitment, which IP3R recruitment signifies, to the cell periphery of cells expressing such mutants indicates that, as is the case for furrow ingression, competency in linking to ESCRT is a property of bud necks formed in the presence of Ca^2+^. 

The ability of the endoplasmic reticulum to form tubules and small vesicles is what permits the stores to be recruited [150]. Movement of IP3R-contaning ER vesicles along microtubules has been shown to be facilitated by a kinesin [151]. Kinesins are a large family of cellular protein motors that use the energy of ATP hydrolysis to induce movement along the microtubule [152]. Kinesins have been identified as being involved in an intracellular process required for Gag release: (i) Kinesin KIF4 was reported to bind Gag directly through the MA domain [153] and was later found to regulate intracellular trafficking and stability of Gag [154]; (ii) Kinesin KIF3, a binding partner of AP-3 shown to be required for release of the viral particles assembled by Gag [72], has also been reported to be involved in Gag release [155]. Which particular kinesin is involved in IP3R transport is unknown. Kinesin-mediated translocation of IP3R along microtubules would allow for directed delivery of Ca^2+^ stores to the budding site and, thereby, establish a localized region where Ca^2+^ would be elevated. Thus, for Ca^2+^ provision, utilization of the internal Ca^2+^ stores may provide a major advantage over Ca^2+^ influx which is mediated by channels that are homogenously distributed on the plasma membrane. 

The notion that intact PI(4,5)P_2_ is required for targeting Gag to the plasma membrane and that PLC-hydrolyzed PI(4,5)P_2_ is required for ESCRT-recruitment-competent bud neck ingression suggests the need for regulatory mechanisms that would ensure availability of the right form of the phospholipid for the right event in the pre-ESCRT stage. The “hydrolysis stimulates synthesis” model proposes that hydrolysis and synthesis of PI(4,5)P_2_ are tightly coupled events such that synthesis stimulates hydrolysis while PI(4,5)P_2_ hydrolysis signals its production [69]. Ca^2+^ might be a key regulator: Ca^2+^ is an activator of the lipid kinase that is critical for PI(4,5)P_2_ synthesis [156] and of the PLC that catalyzes PI(4,5)P_2_ hydrolysis [157]. However, Gag PM targeting appears to require a more nuanced intact PI(4,5)P_2_ population. Although it has been clearly demonstrated that depletion of PI(4,5)P_2_ with plasmamembrane-targeted lipid phosphatases prevents Gag localization to the PM [59], other experimental approaches give different results. For example, increased Gag PM targeting and VLP release were not observed following a clear increase in PM PI(4,5)P_2_ in cells treated with a PLC inhibitor [9]. Also, a loss of Gag PM targeting was reported in cells that did not exhibit a detectable change in PI(4,5)P_2_ level or subcellular distribution [145]. There is growing recognition that PM PI(4,5)P_2_ exists in multiple pools and that the dynamic nature of these pools is important for cellular processes mediated by PI(4,5)P_2_ [148, 156]. Perhaps this conundrum, that is, the lack of a clear correlation between Gag PM targeting and the PI(4,5)P_2_ level, reflects a requirement for a PI(4,5)P_2_ pool that is specifically made available for Gag. The non-ESCRT proteins, Spry2 and ADP-ribosylation factor-1 (ARF1), have activities that make them potential participants in such regulatory mechanisms. Spry2 is required for Gag particle budding [16, 17] and for production of infectious virus (Ehrlich, Khan, Powell and Carter, unpublished observations). It has several activities that can affect PI(4,5)P_2_ metabolism; namely, binding of phospholipase C [13] and of PI(4,5)P_2 _ [13, 17] and it can inhibit receptor-mediated activation of PLC*γ* [13]. Binding to PI(4,5)P_2_ exerted the greatest influence on Gag particle production [17]. Involvement of ARF-1 in Gag assembly was demonstrated by Joshi et al. [145]. Although this protein is best known for its role in post-Golgi trafficking, ARF1 is also a stimulator of PI(4,5)P_2_ synthesis by directly activating PI(4)P 5-kinase and by inducing formation of an enhancer of the kinase [158]. Thus, together with local Ca^2+^, Sprouty and ARF1 proteins have the potential to ensure the dynamic existence of PI(4,5)P_2_ pools specifically made available for interaction with Gag. 

Several other non-ESCRT proteins whose dysfunction inhibited transport of Gag from the cell interior to the plasma membrane may also be involved in Gag assembly as pre-ESCRT stage participants. Admittedly, further studies will be needed to elucidate their exact contribution; however, interestingly, these proteins also have links to cytokinesis and autophagy. In addition to the aforementioned SNARES [145], these include citron kinase, a Rho effector [159]; Rab9 [160] and other GTPases [161]; POSH [162]; AP-1 [144]; NPC-1 [73]; and Filamin A [163]. Direct participation in cytokinesis is documented for citron kinase, AP-1, and Filamin A [164–166]. NPC-1 and POSH both affect the metabolism of two important factors in cytokinesis, cholesterol [167], and calcium [168], respectively. Rab9 and other small GTPases have been implicated in cytokinesis and autophagy [118, 169].

## 7. Non-ESCRT Proteins in the ESCRT Recruitment Stage

 The formation of a Gag-Tsg101 complex occurs as part of the Gag assembly process as long as L domain-1 is intact. Although the precise stage at which Tsg101 docks on the PTAP motif is not known, association after stable bud neck formation might be more favorable as it precludes nonproductive interactions with ESCRT-II that would signal internalization of the Gag assemblage or premature ESCRT-III scission. Spry2 forms complexes with components of ESCRT-II [16]. Thus, Spry2 facilitates release driven by both the primary and the secondary HIV-1 Gag L domains, possibly due to its ability to compete with ESCRT-I factors for interaction with ESCRT-II components [16]. This notion is consistent with the fact that HIV-1 budding does not require ESCRT-II [98, 99]. Not surprisingly since the interaction of ESCRT-I with ESCRT-II leads to cargo internalization, it has been suggested that association with Tsg101 increases susceptibility to internalization [170]. Delaying the recruitment of ESCRT machinery to the budding site may provide a means of maximizing viral budding efficiency. A parallel to this as a regulation possibility in cytokinesis may be the aforementioned function of TEX14, a protein believed to control premature progression to the abscission stage by competing with Tsg101 and Alix for binding to Cep55 [146].

## 8. Concluding Remarks

In this paper, we have focused on proteins involved in steps in HIV-1 trafficking and budding that take place prior to Gag recruitment of ESCRT machinery. As described here, proteins that function in PI(4,5)P_2_ binding, synthesis or hydrolysis, Ca^2+^ store recruitment, IP3R-mediated Ca^2+^ store release, and vesicular biogenesis or transport appear to comprise the major classes of participants in the pre-ESCRT stages. Cellular activities in almost all cells are regulated by common signaling systems and Ca^2+^ is a ubiquitous intracellular messenger that is known to control a diverse range of processes. The discovery of Ca^2+^ signaling as a cofactor in HIV-1 protein trafficking and release, its potential link to exploitation of the ESCRT machinery by the virus for viral particle production, and the general similarity of this coupling to other cellular activities in which ESCRTs participate, that is, cytokinesis and autophagy, may provide new therapeutic avenues for HIV treatment strategies.

## References

[1] Chukkapalli V, Ono A (2011). Molecular determinants that regulate plasma membrane association of HIV-1 Gag. *Journal of Molecular Biology*.

[2] Balasubramaniam M, Freed EO (2011). New insights into HIV assembly and trafficking. *Physiology*.

[3] Bieniasz PD (2009). The cell biology of HIV-1 virion genesis. *Cell Host and Microbe*.

[4] Klein KC, Reed JC, Lingappa JR (2007). Intracellular destinies: degradation, targeting, assembly, and endocytosis of HIV Gag. *AIDS Reviews*.

[5] Goff A, Ehrlich LS, Cohen SN, Carter CA (2003). Tsg101 control of human immunodeficiency virus type 1 Gag trafficking and release. *Journal of Virology*.

[6] Mikoshiba K (2007). The IP3 receptor/Ca^2+^ channel and its cellular function. *Biochemical Society Symposium*.

[7] Patterson RL, Boehning D, Snyder SH (2004). Inositol 1,4,5-trisphosphate receptors as signal integrators. *Annual Review of Biochemistry*.

[8] Vermassen E, Parys JB, Mauger J-P (2004). Subcellular distribution of the inositol 1,4,5-trisphosphate receptors: functional relevance and molecular determinants. *Biology of the Cell*.

[9] Ehrlich LS, Medina GN, Khan MB, Powell MD, Mikoshiba K, Carter CA (2010). Activation of the inositol (1,4,5)-triphosphate calcium gate receptor is required for HIV-1 Gag release. *Journal of Virology*.

[10] Taylor C, Da Fonseca PC, Morris EP (2004). IP3 receptors: the search for structure. *Trends in Biochemical Sciences*.

[11] Perlman M, Resh MD (2006). Identification of an intracellular trafficking and assembly pathway for HIV-1 Gag. *Traffic*.

[12] Grigorov B, Arcanger F, Roingeard P, Darlix JL, Muriaux D (2006). Assembly of infectious HIV-1 in human epithelial and T-lymphoblastic cell lines. *Journal of Molecular Biology*.

[13] Akbulut S, Reddi AL, Aggarwal P (2010). Sprouty proteins inhibit receptor-mediated activation of phosphatidylinositol-specific phospholipase C. *Molecular Biology of the Cell*.

[14] Guy GR, Jackson RA, Yusoff P, Chow SY (2009). Sprouty proteins: modified modulators, matchmakers or missing links?. *Journal of Endocrinology*.

[15] Edwin F, Anderson K, Ying C, Patel TB (2009). Intermolecular interactions of sprouty proteins and their implications in development and disease. *Molecular Pharmacology*.

[16] Medina GN, Ehrlich LS, Chen MH, Khan MB, Powell MD, Carter CA (2011). Sprouty 2 binds ESCRT-II factor Eap20 and facilitates HIV-1 Gag release. *Journal of Virology*.

[17] Ehrlich LS, Medina GN, Carter CA (2011). Sprouty2 regulates PI(4,5)P_2_/Ca^2+^ signaling and HIV-1 Gag release. *Journal of Molecular Biology*.

[18] Chandramouli S, Chye YY, Yusoff P (2008). Tesk1 interacts with Spry2 to abrogate its inhibition of ERK phosphorylation downstream of receptor tyrosine kinase signaling. *Journal of Biological Chemistry*.

[19] Miura GI, Roignant JY, Wassef M, Treisman JE (2008). Myopic acts in the endocytic pathway to enhance signaling by the Drosophila EGF receptor. *Development*.

[20] Kim HJ, Taylor LJ, Bar-Sagi D (2007). Spatial Regulation of EGFR Signaling by Sprouty2. *Current Biology*.

[21] Martin-Serrano J, Neil SJ (2011). Host factors involved in retroviral budding and release. *Nature Reviews Microbiology*.

[22] Chu H, Wang JJ, Spearman P (2009). Human immunodeficiency virus type-1 Gag and host vesicular trafficking pathways. *Current Topics in Microbiology and Immunology*.

[23] Ono A (2009). HIV-1 assembly at the plasma membrane: Gag trafficking and localization. *Future Virology*.

[24] Scarlata S, Carter C (2003). Role of HIV-1 Gag domains in viral assembly. *Biochimica et Biophysica Acta*.

[25] Ono A, Freed EO (1999). Binding of human immunodeficiency virus type 1 Gag to membrane: role of the matrix amino terminus. *Journal of Virology*.

[26] Scarlata S, Ehrlich LS, Carter CA (1998). Membrane-induced alterations in HIV-1 Gag and matrix protein-protein interactions. *Journal of Molecular Biology*.

[27] Ehrlich LS, Fong S, Scarlata S, Zybarth G, Carter C (1996). Partitioning of HIV-1 Gag and Gag-related proteins to membranes. *Biochemistry*.

[28] Zhou W, Parent LJ, Wills JW, Resh MD (1994). Identification of a membrane-binding domain within the amino-terminal region of human immunodeficiency virus type 1 Gag protein which interacts with acidic phospholipids. *Journal of Virology*.

[29] Spearman P, Wang JJ, Heyden NV, Ratner L (1994). Identification of human immunodeficiency virus type 1 Gag protein domains essential to membrane binding and particle assembly. *Journal of Virology*.

[30] Ehrlich LS, Agresta BE, Carter CA (1992). Assembly of recombinant human immunodeficiency virus type 1 capsid protein *in vitro*. *Journal of Virology*.

[31] Campbell S, Vogt VM (1995). Self-assembly *in vitro* of purified CA-NC proteins from Rous sarcoma virus and human immunodeficiency virus type 1. *Journal of Virology*.

[32] Momany C, Kovari LC, Prongay AJ (1996). Crystal structure of dimeric HIV-1 capsid protein. *Nature Structural Biology*.

[33] Gross I, Hohenberg H, Huckhagel C, Kräusslich HG (1998). N-terminal extension of human immunodeficiency virus capsid protein converts the *in vitro* assembly phenotype from tubular to spherical particles. *Journal of Virology*.

[34] Lanman J, Sexton J, Sakalian M, Prevelige PE (2002). Kinetic analysis of the role of intersubunit interactions in human immunodeficiency virus type 1 capsid protein assembly *in vitro*. *Journal of Virology*.

[35] Ganser-Pornillos BK, von Schwedler UK, Stray KM, Aiken C, Sundquist WI (2004). Assembly properties of the human immunodeficiency virus type 1 CA protein. *Journal of Virology*.

[36] Mateu MG (2009). The capsid protein of human immunodeficiency virus: intersubunit interactions during virus assembly. *FEBS Journal*.

[37] Gorelick RJ, Chabot DJ, Rein A, Henderson LE, Arthur LO (1993). The two zinc fingers in the human immunodeficiency virus type 1 nucleocapsid protein are not functionally equivalent. *Journal of Virology*.

[38] De Guzman RN, Wu ZR, Stalling CC, Pappalardo L, Borer PN, Summers MF (1998). Structure of the HIV-1 nucleocapsid protein bound to the SL3 *ψ*-RNA recognition element. *Science*.

[39] Lingappa JR, Dooher JE, Newman MA, Kiser PK, Klein KC (2006). Basic residues in the nucleocapsid domain of Gag are required for interaction of HIV-1 Gag with ABCE1 (HP68), a cellular protein important for HIV-1 capsid assembly. *Journal of Biological Chemistry*.

[40] Muriaux D, Darlix JL (2010). Properties and functions of the nucleocapsid protein in virus assembly. *RNA Biology*.

[41] Gottlinger HG, Dorfman T, Sodroski JG, Haseltine WA (1991). Effect of mutations affecting the p6 Gag protein on human immunodeficiency virus particle release. *Proceedings of the National Academy of Sciences of the United States of America*.

[42] Huang M, Orenstein JM, Martin MA, Freed EO (1995). p6Gag is required for particle production from full-length human immunodeficiency virus type 1 molecular clones expressing protease. *Journal of Virology*.

[43] Pettit SC, Lindquist JN, Kaplan AH, Swanstrom R (2005). Processing sites in the human immunodeficiency virus type 1 (HIV-1) Gag-Pro-Pol precursor are cleaved by the viral protease at different rates. *Retrovirology*.

[44] Zybarth G, Carter C (1995). Domains upstream of the protease (PR) in human immunodeficiency virus type 1 Gag-Pol influence PR autoprocessing. *Journal of Virology*.

[45] Zybarth G, Krausslich HG, Partin K, Carter C (1994). Proteolytic activity of novel human immunodeficiency virus type 1 proteinase proteins from a precursor with a blocking mutation at the N terminus of the PR domain. *Journal of Virology*.

[46] Partin K, Zybarth G, Ehrlich L, DeCrombrugghe M, Wimmer E, Carter C (1991). Deletion of sequences upstream of the proteinase improves the proteolytic processing of human immunodeficiency virus type 1. *Proceedings of the National Academy of Sciences of the United States of America*.

[47] Partin K, Wimmer E, Carter C (1991). Mutational analysis of a native substrate of the HIV-1 proteinase. *Advances in Experimental Medicine and Biology*.

[48] Sakuragi J (2011). Morphogenesis of the infectious HIV-1 virion. *Frontiers in Microbiology*.

[49] Briggs JA, Krausslich HG (2011). The molecular architecture of HIV. *Journal of Molecular Biology*.

[50] Bieniasz PD (2006). Late budding domains and host proteins in enveloped virus release. *Virology*.

[51] Freed EO (2002). Viral late domains. *Journal of Virology*.

[52] Wills JW, Craven RC (1991). Form, function, and use of retroviral Gag proteins. *AIDS*.

[53] Potter MD, Seiser RM, Nicchitta CV (2001). Ribosome exchange revisited: a mechanism for translation-coupled ribosome detachment from the ER membrane. *Trends in Cell Biology*.

[54] Bryant M, Ratner L (1990). Myristoylation-dependent replication and assembly of human immunodeficiency virus 1. *Proceedings of the National Academy of Sciences of the United States of America*.

[55] Gottlinger HG, Sodroski JG, Haseltine WA (1989). Role of capsid precursor processing and myristoylation in morphogenesis and infectivity of human immunodeficiency virus type 1. *Proceedings of the National Academy of Sciences of the United States of America*.

[56] Tritel M, Resh MD (2000). Kinetic analysis of human immunodeficiency virus type 1 assembly reveals the presence of sequential intermediates. *Journal of Virology*.

[57] Nermut MV, Zhang WH, Francis G, Čiampor F, Morikawa Y, Jones IM (2003). Time course of Gag protein assembly in HIV-1-infected cells: a study by immunoelectron microscopy. *Virology*.

[58] Aloia RC, Tian H, Jensen FC (1993). Lipid composition and fluidity of the human immunodeficiency virus envelope and host cell plasma membranes. *Proceedings of the National Academy of Sciences of the United States of America*.

[59] Ono A, Ablan SD, Lockett SJ, Nagashima K, Freed EO (2004). Phosphatidylinositol (4,5) bisphosphate regulates HIV-1 Gag targeting to the plasma membrane. *Proceedings of the National Academy of Sciences of the United States of America*.

[60] Watt SA, Kular G, Fleming IN, Downes CP, Lucocq JM (2002). Subcellular localization of phosphatidylinositol 4,5-bisphosphate using the pleckstrin homology domain of phospholipase C *δ*1. *Biochemical Journal*.

[61] Saad JS, Miller J, Tai J, Kim A, Ghanam RH, Summers MF (2006). Structural basis for targeting HIV-1 Gag proteins to the plasma membrane for virus assembly. *Proceedings of the National Academy of Sciences of the United States of America*.

[62] Anraku K, Fukuda R, Takamune N (2010). Highly sensitive analysis of the interaction between HIV-1 Gag and phosphoinositide derivatives based on surface plasmon resonance. *Biochemistry*.

[63] Shkriabai N, Datta SA, Zhao Z, Hess S, Rein A, Kvaratskhelia M (2006). Interactions of HIV-1 Gag with assembly cofactors. *Biochemistry*.

[64] Chukkapalli V, Hogue IB, Boyko V, Hu WS, Ono A (2008). Interaction between the human immunodeficiency virus type 1 Gag matrix domain and phosphatidylinositol-(4,5)-bisphospnate is essential for efficient Gag membrane binding. *Journal of Virology*.

[65] Fernandes F, Chen K, Ehrlich LS (2011). Phosphoinositides direct equine infectious anemia virus Gag trafficking and release. *Traffic*.

[66] Hamard-Peron E, Juillard F, Saad JS (2010). Targeting of murine leukemia virus Gag to the plasma membrane is mediated by PI(4,5)P_2_/PS and a polybasic region in the matrix. *Journal of Virology*.

[67] Dove SK, Dong K, Kobayashi T, Williams FK, Michell RH (2009). Phosphatidylinositol 3,5-bisphosphate and Fab1p/PIKfyve underPPIn endo-lysosome function. *Biochemical Journal*.

[68] Chan J, Dick RA, Vogt VM (2011). Rous sarcoma virus Gag has no specific requirement for phosphatidylinositol-(4,5)-bisphosphate for plasma membrane association *in vivo* or for liposome interaction *in vitro*. *Journal of Virology*.

[69] Loew LM (2007). Where does all the PIP_2_ come from?. *Journal of Physiology*.

[70] Sherer NM, Lehmann MJ, Jimenez-Soto LF (2003). Visualization of retroviral replication in living cells reveals budding into multivesicular bodies. *Traffic*.

[71] Raposo G, Moore M, Innes D (2002). Human macrophages accumulate HIV-1 particles in MHC II compartments. *Traffic*.

[72] Dong X, Li H, Derdowski A (2005). AP-3 directs the intracellular trafficking of HIV-1 Gag and plays a key role in particle assembly. *Cell*.

[73] Tang Y, Leao IC, Coleman EM, Broughton RS, Hildreth JEK (2009). Deficiency of niemann-pick type C-1 protein impairs release of human immunodeficiency virus type 1 and results in Gag accumulation in late endosomal/lysosomal compartments. *Journal of Virology*.

[74] Nguyen DG, Booth A, Gould SJ, Hildreth JEK (2003). Evidence that HIV budding in primary macrophages occurs through the exosome release pathway. *Journal of Biological Chemistry*.

[75] Deneka M, Pelchen-Matthews A, Byland R, Ruiz-Mateos E, Marsh M (2007). In macrophages, HIV-1 assembles into an intracellular plasma membrane domain containing the tetraspanins CD81, CD9, and CD53. *Journal of Cell Biology*.

[76] Welsch S, Keppler OT, Habermann A, Allespach I, Krijnse-Locker J, Kräusslich HG (2007). HIV-1 buds predominantly at the plasma membrane of primary human macrophages. *PLoS Pathogens*.

[77] Jouvenet N, Neil SJD, Bess C (2006). Plasma membrane is the site of productive HIV-1 particle assembly. *PLoS Biology*.

[78] Ono A, Freed EO (2004). Cell-type-dependent targeting of human immunodeficiency virus type 1 assembly to the plasma membrane and the multivesicular body. *Journal of Virology*.

[79] Xiang Y, Cameron CE, Wills JW, Leis J (1996). Fine mapping and characterization of the Rous sarcoma virus Pr76(Gag) late assembly domain. *Journal of Virology*.

[80] Parent LJ, Bennett RP, Craven RC (1995). Positionally independent and exchangeable late budding functions of the rous sarcoma virus and human immunodeficiency virus Gag proteins. *Journal of Virology*.

[81] Yuan B, Campbell S, Bacharach E, Rein A, Goff SP (2000). Infectivity of Moloney murine leukemia virus defective in late assembly events is restored by late assembly domains of other retroviruses. *Journal of Virology*.

[82] Li F, Chen C, Puffer BA, Montelaro RC (2002). Functional replacement and positional dependence of homologous and heterologous L domains in equine infectious anemia virus replication. *Journal of Virology*.

[83] Ott DE, Coren LV, Gagliardi TD, Nagashima K (2005). Heterologous late-domain sequences have various abilities to promote budding of human immunodeficiency virus type 1. *Journal of Virology*.

[84] Carter CA (2002). Tsg101: HIV-1’s ticket to ride. *Trends in Microbiology*.

[85] Pincetic A, Leis J (2009). The mechanism of budding of retroviruses from cell membranes. *Advances in Virology*.

[86] Medina G, Zhang Y, Tang Y (2005). The functionally exchangeable L domains in RSV and HIV-1 Gag direct particle release through pathways linked by Tsg101. *Traffic*.

[87] Medina G, Pincetic A, Ehrlich LS (2008). Tsg101 can replace Nedd4 function in ASV Gag release but not membrane targeting. *Virology*.

[88] Chung HY, Morita E, von Schwedler U, Muller B, Krausslich HG, Sundquist WI (2008). NEDD4L overexpression rescues the release and infectivity of human immunodeficiency virus type 1 constructs lacking PTAP and YPXL late domains. *Journal of Virology*.

[89] Usami Y, Popov S, Popova E, Göttlinger HG (2008). Efficient and specific rescue of human immunodeficiency virus type 1 budding defects by a Nedd4-like ubiquitin ligase. *Journal of Virology*.

[90] Weiss ER, Popova E, Yamanaka H, Kim HC, Huibregtse JM, Gottlinger H (2010). Rescue of HIV-1 release by targeting widely divergent NEDD4-type ubiquitin ligases and isolated catalytic HECT domains to Gag. *PLoS Pathogens*.

[91] Sette P, Jadwin JA, Dussupt V, Bello NF, Bouamr F (2010). The ESCRT-associated protein alix recruits the ubiquitin ligase Nedd4-1 to facilitate HIV-1 release through the LYPXnL L domain motif. *Journal of Virology*.

[92] Fujii K, Hurley JH, Freed EO (2007). Beyond Tsg101: the role of alix in “ESCRTing” HIV-1. *Nature Reviews Microbiology*.

[93] Henne WM, Buchkovich NJ, Emr SD (2011). The ESCRT pathway. *Developmental Cell*.

[94] Hurley JH (2010). The ESCRT complexes. *Critical Reviews in Biochemistry and Molecular Biology*.

[95] Roxrud I, Stenmark H, Malerød L (2010). ESCRT & Co. *Biology of the Cell*.

[96] Adell MA, Teis D (2011). Assembly and disassembly of the ESCRT-III membrane scission complex. *FEBS Letters*.

[97] Hurley JH, Hanson PI (2010). Membrane budding and scission by the ESCRT machinery: it’s all in the neck. *Nature Reviews Molecular Cell Biology*.

[98] Langelier C, von Schwedler UK, Fisher RD (2006). Human ESCRT-II complex and its role in human immunodeficiency virus type 1 release. *Journal of Virology*.

[99] Pincetic A, Medina G, Carter C, Leis J (2008). Avian sarcoma virus and human immunodeficiency virus, type 1 use different subsets of ESCRT proteins to facilitate the budding process. *Journal of Biological Chemistry*.

[100] Ceruti L, Simanis V (2000). Controlling the end of the cell cycle. *Current Opinion in Genetics & Development*.

[101] Carlton JG, Martin-Serrano J (2007). Parallels between cytokinesis and retroviral budding: a role for the ESCRT machinery. *Science*.

[102] Morita E, Sandrin V, Chung HY (2007). Human ESCRT and ALIX proteins interact with proteins of the midbody and function in cytokinesis. *The EMBO Journal*.

[103] Capiod T (2011). Cell proliferation, calcium influx and calcium channels. *Biochemie*.

[104] Clapham DE (2007). Calcium signaling. *Cell*.

[105] Smyth JT, Hwang SY, Tomita T, DeHaven WI, Mercer JC, Putney JW (2010). Activation and regulation of store-operated calcium entry. *Journal of Cellular and Molecular Medicine*.

[106] Vaca L (2010). SOCIC: the store-operated calcium influx complex. *Cell Calcium*.

[107] Arredouani A, Yu F, Sun L, Machaca K (2010). Regulation of store-operated Ca^2+^ entry during the cell cycle. *Journal of Cell Science*.

[108] Smyth JT, Petranka JG, Boyles RR (2009). Phosphorylation of STIM1 underlies suppression of store-operated calcium entry during mitosis. *Nature cell biology*.

[109] Bootman MD, Collins TJ, Mackenzie L, Roderick HL, Berridge MJ, Peppiatt CM (2002). 2-Aminoethoxydiphenyl borate (2-APB) is a reliable blocker of store-operated Ca^2+^ entry but an inconsistent inhibitor of InsP3-induced Ca^2+^ release. *The FASEB Journal*.

[110] Ehrlich LS, Medina GN, Carter CA (2011). ESCRT machinery potentiates HIV-1 utilization of the PI(4,5)P(2)-PLC-IP3R-Ca^2+^ signaling cascade. *Journal of Molecular Biology*.

[111] Hubbard K, Hepler JR (2006). Cell signalling diversity of the Gq*α* family of heterotrimeric G proteins. *Cellular Signalling*.

[112] Banerjee S, Hasan G (2005). The InsP3 receptor: its role in neuronal physiology and neurodegeneration. *BioEssays*.

[113] Ito J, Yoon SY, Lee B (2008). Inositol 1,4,5-trisphosphate receptor 1, a widespread Ca^2+^ channel, is a novel substrate of polo-like kinase 1 in eggs. *Developmental Biology*.

[114] Li WM, Webb SE, Chan CM, Miller AL (2008). Multiple roles of the furrow deepening Ca^2+^ transient during cytokinesis in zebrafish embryos. *Developmental Biology*.

[115] Naito Y, Okada M, Yagisawa H (2006). Phospholipase C isoforms are localized at the cleavage furrow during cytokinesis. *Journal of Biochemistry*.

[116] Wong R, Hadjiyanni I, Wei HC (2005). PIP2 hydrolysis and calcium release are required for cytokinesis in drosophila spermatocytes. *Current Biology*.

[117] Wong R, Fabian L, Forer A, Brill JA (2007). Phospholipase C and myosin light chain kinase inhibition define a common step in actin regulation during cytokinesis. *BMC Cell Biology*.

[118] Dambournet D, MacHicoane M, Chesneau L (2011). Rab35 GTPase and OCRL phosphatase remodel lipids and F-actin for successful cytokinesis. *Nature Cell Biology*.

[119] Mitsuyama F, Futatsugi Y, Okuya M (2008). Microinjected F-actin into dividing newt eggs moves toward the next cleavage furrow together with Ca^2+^ stores with inositol 1,4,5-trisphospnate receptor in a microtubule- and microtubule motor- dependent manner. *Italian Journal of Anatomy and Embryology*.

[120] Almirza WH, Peters PH, van Zoelen EJ, Theuvenet AP (2012). Role of Trpc channels, Stim1 and Orai1 in PGF(2*α*)-induced calcium signaling in NRK fibroblasts. *Cell Calcium*.

[121] Liao Y, Plummer NW, George MD, Abramowitz J, Zhu MX, Birnbaumer L (2009). A role for Orai in TRPC-mediated Ca^2+^ entry suggests that a TRPC:Orai complex may mediate store and receptor operated Ca^2+^ entry. *Proceedings of the National Academy of Sciences of the United States of America*.

[122] Fabbro M, Zhou BB, Takahashi M (2005). Cdk1/Erk2- and Plk1-dependent phosphorylation of a centrosome protein, Cep55, is required for its recruitment to midbody and cytokinesis. *Developmental Cell*.

[123] Martinez-Garay I, Rustom A, Gerdes HH, Kutsche K (2006). The novel centrosomal associated protein CEP55 is present in the spindle midzone and the midbody. *Genomics*.

[124] Lee HH, Elia N, Ghirlando R, Lippincott-Schwartz J, Hurley JH (2008). Midbody targeting of the ESCRT machinery by a noncanonical coiled coil in CEP55. *Science*.

[125] Neto H, Gould GW (2011). The regulation of abscission by multi-protein complexes. *Journal of Cell Science*.

[126] Caballe A, Martin-Serrano J (2011). ESCRT machinery and cytokinesis: the road to daughter cell separation. *Traffic*.

[127] Elia N, Sougrat R, Spurlin TA, Hurley JH, Lippincott-Schwartz J (2011). Dynamics of endosomal sorting complex required for transport (ESCRT) machinery during cytokinesis and its role in abscission. *Proceedings of the National Academy of Sciences of the United States of America*.

[128] Mizushima N, Komatsu M (2011). Autophagy: renovation of cells and tissues. *Cell*.

[129] Kuo TC, Chen CT, Baron D (2011). Midbody accumulation through evasion of autophagy contributes to cellular reprogramming and tumorigenicity. *Nature Cell Biology*.

[130] Rusten TE, Vaccari T, Stenmark H (2011). Shaping development with ESCRTs. *Nature Cell Biology*.

[131] Rusten TE, Simonsen A (2008). ESCRT functions in autophagy and associated disease. *Cell Cycle*.

[132] Metcalf D, Isaacs AM (2010). The role of ESCRT proteins in fusion events involving lysosomes, endosomes and autophagosomes. *Biochemical Society Transactions*.

[133] Decuypere JP, Welkenhuyzen K, Luyten T (2011). Ins(1,4,5)P3 receptor-mediated Ca^2+^ signaling and autophagy induction are interrelated. *Autophagy*.

[134] Girardi JP, Pereira L, Bakovic M (2011). De novo synthesis of phospholipids is coupled with autophagosome formation. *Medical Hypotheses*.

[135] Shen D, Wang X, Xu H (2011). Pairing phosphoinositides with calcium ions in endolysosomal dynamics: phosphoinositides control the direction and specificity of membrane trafficking by regulating the activity of calcium channels in the endolysosomes. *BioEssays*.

[136] Chen Y, Gan BQ, Tang BL (2010). Syntaxin 16: unraveling cellular physiology through a ubiquitous SNARE molecule. *Journal of Cellular Physiology*.

[137] Joshi A, Garg H, Ablan SD, Freed EO (2011). Evidence of a role for soluble N-ethylmaleimide-sensitive factor attachment protein receptor (SNARE) machinery in HIV-1 assembly and release. *The Journal of Biological Chemistry*.

[138] Nair U, Klionsky DJ (2011). Autophagosome biogenesis requires SNAREs. *Autophagy*.

[139] Stroupe C (2011). Autophagy: cells SNARE selves. *Current Biology*.

[140] Dordor A, Poudevigne E, Gottlinger H, Weissenhorn W (2011). Essential and supporting host cell factors for HIV-1 budding. *Future Microbiology*.

[141] Tomita Y, Noda T, Fujii K, Watanabe T, Morikawa Y, Kawaoka Y (2011). The cellular factors Vps18 and Mon2 are required for efficient production of infectious HIV-1 particles. *Journal of Virology*.

[142] Wang G, Deschenes RJ (2006). Plasma membrane localization of ras requires class C Vps proteins and functional mitochondria in saccharomyces cerevisiae. *Molecular and Cellular Biology*.

[143] Bugnicourt A, Froissard M, Sereti K, Ulrich HD, Haguenauer-Tsapis R, Galan JM (2004). Antagonistic roles of ESCRT and Vps class C/HOPS complexes in the recycling of yeast membrane proteins. *Molecular Biology of the Cell*.

[144] Camus G, Segura-Morales C, Molle D (2007). The clathrin adaptor complex AP-1 binds HIV-1 and MLV Gag and facilitates their budding. *Molecular Biology of the Cell*.

[145] Joshi A, Garg H, Nagashima K, Bonifacino JS, Freed EO (2008). GGA and arf proteins modulate retrovirus assembly and release. *Molecular Cell*.

[146] Iwamori T, Iwamori N, Ma L, Edson MA, Greenbaum MP, Matzuk MM (2010). TEX14 interacts with CEP55 to block cell abscission. *Molecular and Cellular Biology*.

[147] Chan R, Uchil PD, Jin J (2008). Retroviruses human immunodeficiency virus and murine leukemia virus are enriched in phosphoinositides. *Journal of Virology*.

[148] McLaughlin S, Murray D (2005). Plasma membrane phosphoinositide organization by protein electrostatics. *Nature*.

[149] Levental I, Christian DA, Wang YH, Madara JJ, Discher DE, Janmey PA (2009). Calcium-dependent lateral organization in phosphatidylinositol 4,5-bisphosphate (PIP2)- and cholesterol-containing monolayers. *Biochemistry*.

[150] Pendin D, McNew JA, Daga A (2011). Balancing ER dynamics: shaping, bending, severing, and mending membranes. *Current Opinion in Cell Biology*.

[151] Bannai H, Inoue T, Nakayama T, Hattori M, Mikoshiba K (2004). Kinesin dependent, rapid, bi-directional transport of ER sub-compartment in dendrites of hippocampal neurons. *Journal of Cell Science*.

[152] Verhey KJ, Kaul N, Soppina V (2011). Kinesin assembly and movement in cells. *Annual Review of Biophysics*.

[153] Tang Y, Winkler U, Freed EO (1999). Cellular motor protein KIF-4 associates with retroviral Gag. *Journal of Virology*.

[154] Martinez NW, Xue X, Berro RG, Kreitzer G, Resh MD (2008). Kinesin KIF4 regulates intracellular trafficking and stability of the human immunodeficiency virus type 1 Gag polyprotein. *Journal of Virology*.

[155] Azevedo C, Burton A, Ruiz-Mateos E, Marsh M, Saiardi A (2009). Inositol pyrophosphate mediated pyrophosphorylation of AP3B1 regulates HIV-1 Gag release. *Proceedings of the National Academy of Sciences of the United States of America*.

[156] Doughman RL, Firestone AJ, Anderson RA (2003). Phosphatidylinositol phosphate kinases put PI4,5P_2_ in its place. *Journal of Membrane Biology*.

[157] Suh PG, Park JI, Manzoli L (2008). Multiple roles of phosphoinositide-specific phospholipase C isozymes. *Journal of Biochemistry and Molecular Biology*.

[158] Skippen A, Jones DH, Morgan CP, Li M, Cockcroft S (2002). Mechanism of ADP ribosylation factor-stimulated phosphatidylinositol 4,5-bisphosphate synthesis in HL60 cells. *Journal of Biological Chemistry*.

[159] Loomis RJ, Holmes DA, Elms A, Solski PA, Der CJ, Su L (2006). Citron kinase, a RhoA effector, enhances HIV-1 virion production by modulating exocytosis. *Traffic*.

[160] Murray JL, Mavrakis M, McDonald NJ (2005). Rab9 GTPase is required for replication of human immunodeficiency virus type 1, filoviruses, and measles virus. *Journal of Virology*.

[161] Audoly G, Popoff MR, Gluschankof P (2005). Involvement of a small GTP binding protein in HIV-I release. *Retrovirology*.

[162] Alroy I, Tuvia S, Greener T (2005). The trans-Golgi network-associated human ubiquitin-protein ligase POSH is essential for HIV type 1 production. *Proceedings of the National Academy of Sciences of the United States of America*.

[163] Cooper J, Liu L, Woodruff EA (2011). Filamin a protein interacts with human immunodeficiency virus type 1 Gag protein and contributes to productive particle assembly. *The Journal of Biological Chemistry*.

[164] Gai M, Camera P, Dema A (2011). Citron kinase controls abscission through RhoA and anilli. *Molecular Biology of the Cell*.

[165] Kita A, Sugiura R, Shoji H (2004). Loss of Apm1, the *μ*1 subunit of the clathrin-associated adaptor-protein-1 complex, causes distinct phenotypes and synthetic lethality with calcineurin deletion in fission yeast. *Molecular Biology of the Cell*.

[166] Mondal S, Burgute B, Rieger D (2010). Regulation of the actin cytoskeleton by an interaction of IQGAP related protein GAPA with filamin and cortexillin I. *PLoS One*.

[167] Fernandez C, Lobo MMV, Gomez-Coronado D, Lasuncion MA (2004). Cholesterol is essential for mitosis progression and its deficiency induces polyploid cell formation. *Experimental Cell Research*.

[168] Tuvia S, Taglicht D, Erez O (2007). The ubiquitin E3 ligase POSH regulates calcium homeostasis through spatial control of Herp. *Journal of Cell Biology*.

[169] Chua CE, Gan BQ, Tang BL (2011). Involvement of members of the Rab family and related small GTPases in autophagosome formation and maturation. *Cellular and Molecular Life Sciences*.

[170] Harila K, Prior I, Sjoberg M, Salminen A, Hinkula J, Suomalainen M (2006). Vpu and Tsg101 regulate intracellular targeting of the human immunodeficiency virus type 1 core protein precursor Pr55gag. *Journal of Virology*.

